# Gallstone-Associated Histopathological Changes in Liver: A Prospective Observational Study

**DOI:** 10.7759/cureus.55417

**Published:** 2024-03-02

**Authors:** Rinku Kumari, Rajeev Ranjan, Pradeep Jaiswal, Pawan K Jha, Kancham Nethaji, Ankur Akela

**Affiliations:** 1 Department of General Surgery, Indira Gandhi Institute of Medical Sciences, Patna, IND; 2 Department of Urology, Indira Gandhi Institute of Medical Sciences, Patna, IND

**Keywords:** liver biopsy, liver histology, gall stone disease, choledocholithiasis, cholelithiasis

## Abstract

Aim and Objectives: To assess the effect of gallstone disease on liver parenchyma and the prevalence and extent of liver pathology in cholelithiasis in our population at the Department of General Surgery, Indira Gandhi Institute of Medical Science (IGIMS), Patna.

Material and Methods: The present prospective observational study was conducted on 100 either-sex patients scheduled for open or laparoscopic cholecystectomy. In all the patients, laboratory and radiological investigations were performed. An undamaged portion of the liver edge around the gallbladder fossa was selected and held by atraumatic forceps. Using sharp scissors, around 1 cm of the liver edge was taken out and sent for histopathological examination.

Results: The mean age of the patients was 39.28 ± 13.73 years. The majority of patients were females (69%). Pain was the predominant clinical feature in 51% of the patients, followed by vomiting (21%), nausea (18%), and indigestion (10%). In 36% of cases, the liver histology was abnormal, including steatosis, fibrosis, cholestasis, portal tract infiltration, and lobular parenchymal infiltration. A significant association was found between the duration of symptoms and abnormal histology findings (P<0.0001).

Conclusion: Gallstone disease is associated with notable alterations in liver histology, and these changes tend to be more prevalent in individuals with a prolonged duration of symptoms.

## Introduction

Gallstones affect 3-6% of adult Indians and 10-15% of the general adult populace [1]. Cholecystectomy is one of the utmost frequent procedures carried out by general surgeons because 1-4% of people experience symptoms every year that necessitate it [1]. Gallstones form when substances in bile reach a point where they can no longer dissolve. As bile concentration increases in the gallbladder, it becomes supersaturated with these substances, leading to the gradual precipitation of small crystals. These crystals then adhere to the gallbladder mucus, giving rise to gallbladder sludge. Over time, these crystals enlarge and coalesce to form larger stones. Complications arising from gallstones result from the obstruction of the hepatic and biliary tree by sludge and stones [2].

Patients with gallstone disease may develop cholangitis, a condition that, over time, can lead to secondary hepatic changes such as fatty liver, fibrosis, secondary biliary cirrhosis, reactive hepatitis, and nonspecific inflammatory alterations. Notably, individuals with choledocholithiasis experience more extensive liver damage compared to those with cholecystitis and cholelithiasis [3].

The occurrence of hepatocellular damage in patients with gallstone disease has been a topic of debate for many years [4]. As early as 1918, Graham was the first to highlight the potential influence of gallstone disease on the liver, emphasizing the necessity for thorough examination [5]. Subsequently, between 1942 and 1948, Mateer et al. conducted pioneering research that became a crucial milestone in comprehending this association [6]. However, later studies by Keller and Smetana [7] and Edlund and Zettergren [8] presented contrasting viewpoints, challenging Mateer's assertion that cholelithiasis and cholecystitis lead to significant histological liver damage [7,8]. In contrast, researchers such as Dunlap et al. [9,10], Savory et al. [11], and Raven [12] supported Mateer et al.'s findings [6]. This divergence in perspectives underscores the ongoing exploration and refinement of our understanding of the relationship between gallstone disease and its potential impact on liver histology.

Evidence has demonstrated that patients with cholangitis, resulting from various causes leading to partial obstruction of the extra-hepatic large bile duct, exhibit both structural and functional alterations in the liver [13,14]. These changes may diminish upon the removal of the obstruction, restoring bile flow within the biliary ductal system and resolving the inflammatory process. This suggests a direct correlation between the degree of liver inflammation and obstruction in the biliary tree [14]. Previous studies have noted a minor degree of fibrosis in portal tracts, with conflicting results regarding the association of gallstones with changes in liver histology, and cholestasis was not consistently emphasized [13,14]. A recent study in India found that the most frequently observed liver histological changes in cholelithiasis patients were nonspecific reactive hepatitis (46.9%), while in choledocholithiasis, chronic cholestasis was predominant (50%). Patients with choledocholithiasis exhibited significant changes (p<0.001) in liver histopathology and liver function tests compared to those with cholelithiasis [15].

This present study aimed to assess the effect of gallstone disease on liver parenchyma and the prevalence and extent of liver pathology in cholelithiasis in the population at the Department of General Surgery, Indira Gandhi Institute of Medical Science (IGIMS), Patna.

## Materials and methods

The present prospective observational study was conducted at the Department of General Surgery, IGIMS in Sheikhpura, Patna, from December 2020 to December 2022, following approval from the institutional ethical committee. A total of 100 participants of either gender, with signs and symptoms of gallstone disease and common bile duct stones, scheduled for open or laparoscopic cholecystectomy were recruited for the study. Patients with a recent history of jaundice, use of hepatotoxic medication, portal hypertension, hepatitis B or C, altered coagulation profile, acute cholecystitis, and fatty liver were excluded from the study.

Before enrollment, a comprehensive explanation of the study procedure was provided to the patients and their relatives. Written informed consent was obtained from all subjects. Sociodemographic data of patients was obtained and were clinically and physically assessed. Subsequently, laboratory and radiological investigations were conducted for each participant. Both open and laparoscopic techniques were employed for gallbladder aspiration and cholecystectomy.

Following gallbladder removal, hemostasis was carefully achieved around the gallbladder fossa. The process of achieving hemostasis around the gallbladder fossa involved the use of electrocautery to ensure a controlled and effective approach.

To collect specimens for histopathological examination, an undamaged portion of the liver edge around the gallbladder fossa was selected. Atraumatic forceps were used to hold the selected portion, and approximately 1 cm of the liver edge was excised using sharp scissors. The excised liver tissue was then sent for histopathological examination.

Statistical analysis

Data were recorded in Microsoft® Excel Worksheet 2019 and exported into SPSS v21.0 (International Business Machines, New York, United States) for statistical analysis. Quantitative data were expressed as mean and standard deviation (SD). Categorical data were expressed as frequency and percentages and compared using the chi-square test. P<0.05 was considered statistically significant.

## Results

The mean age of the patients was 39.28 ± 13.73 years. The majority of patients were females (69%) (Table 1). Out of the total number of patients in the study, six patients had diabetes mellitus and 15 patients had a history of hypertension. The female patients did not have a history of polycystic ovarian syndrome.

**Table 1:** Distribution of subjects according to demography BMI: Body mass index

Variables	Subcategories	Frequency
Age (years)	<20	3
	21-30	21
	31-40	28
	41-50	31
	51-60	10
	>60	7
Sex	Male	31
	Female	69
Socioeconomic status	Lower	36
	Middle	37
	Upper	27
BMI (kg/m^2^)	Underweight	13
	Normal	54
	Overweight	21
	Obese	12
Substance abuse	Smoking	7
	Alcohol	8

Pain was the predominant clinical feature in 51% of the patients, followed by vomiting (21%), nausea (18%), and indigestion (10%). In this study, liver histology was normal in 64% of the patients. In 36% of the patients, liver histology was abnormal. Abnormal histology involved portal tract infiltration (38.9%), steatosis (30.6%), cholestasis (13.9%), lobular parenchymal infiltration (13.9%), and fibrosis (2.8%) (Table 2). Steatosis was confirmed after a histopathological examination.

**Table 2:** Distribution of subjects according to abnormal liver histology findings

Abnormal liver histology	Frequency (n)	Percentage (%)
Portal tract infiltration	14	38.9
Steatosis	11	30.6
Cholestasis	5	13.9
Lobular parenchymal infiltration	5	13.9
Fibrosis	1	2.8
Total	36	100

A significant association was found between the duration of symptoms and abnormal histology findings (P<0.0001) (Table 3). Age, gender, smoking, alcohol abuse, hypertension, diabetes, presence of multiple stones, and BMI were not associated with abnormal liver histology for which p values are statistically not significant. 

**Table 3:** Association between study variables and abnormal liver histology BMI: Body mass index

Variables	Subcategories	Liver Histology		P-value
		Abnormal	Normal	
Age (years)	<20	0	3	0.395
	21-30	6	15	
	31-40	12	16	
	41-50	14	17	
	51-60	2	8	
	>60	2	5	
Sex	Male	7	24	0.393
	Female	29	40	
BMI (kg/m^2^)	Underweight	5	8	0.083
	Normal	14	40	
	Overweight	12	9	
	Obese	5	7	
Substance abuse	Smoking	4	3	0.180
	Alcohol	4	4	0.319
Duration of symptoms (months)	0-6	1	33	<0.0001
	7-12	13	11	
	13-24	12	10	
	25-36	10	10	
	>36	0	0	
Comorbidities	Hypothyroidism	4	5	0.300
	Hypotension	8	7	
	Diabetes	3	3	
Number of gallstones	Single	10	10	0.099
	Multiple	26	54	

## Discussion

Liver damage in individuals with gallstones is believed to stem from prolonged obstruction of the large extrahepatic bile duct, with or without recurrent episodes of cholangitis, potentially advancing to secondary biliary cirrhosis. Previous studies examining the connection between gallstone presence and alterations in liver histology have yielded conflicting results. Notably, reported incidences of portal tract fibrosis have shown significant variation, and the relationship between fibrosis and the localization of gallstones has been inadequately characterized. Importantly, detailed descriptions of the histological changes have been lacking. The study aimed to assess the effect of gallstone disease on liver parenchyma and the prevalence and extent of liver pathology in cholelithiasis in the population at the Department of General Surgery, IGIMS, Patna. In this study, a total of 100 patients scheduled for laparoscopic cholecystectomy were assessed.

The traditional risk factors for gallstone disease are known to be 4F: fat, female, forty, and fertile. Similarly, in this study, the mean age of the patients was 39.28 ± 13.73 years, and most patients were female. These findings are similar to the previous reports [3,4,16,17].

In this study, more than half of the patients (51%) had pain followed by vomiting (21%), nausea (18%), and indigestion (10%). There were 15% hypertensive, 9% had hypothyroidism, and 6% had diabetes. None of the patients had a symptom duration of more than 36 months, while 34% had symptoms of up to six months. After the gallbladder was taken out, hemostasis was achieved around the gallbladder fossa. An undamaged portion of the liver edge was selected and held by atraumatic forceps. With the help of sharp scissors, around 1 cm of liver edge was taken out and sent for histopathological evaluation. In 64 patients, liver biopsy was normal, but in 36 patients, significant changes were present in liver histology such as portal tract infiltration (38.9%), steatosis (30.6%), cholestasis (13.9%), lobular parenchymal infiltration (13.9%), and fibrosis (2.8%). Steatosis was due to gallstone since ultrasonography showed no pathology in the liver, and clinically, there were no signs of liver cell failure. Alcoholic patients with fatty liver on ultrasound were excluded from the study. Portal tracts consist of a bile duct, portal vein, and arteriole surrounded by a collagenous matrix. Blood flows to the liver through the portal tract while blood exits the liver through central veins. Portal tract infiltration was due to portal inflammation, which comprised of cells referred to as chronic (i.e., lymphocytes, plasma cells, occasional eosinophils, and monocytes).

The inclusion of symptom duration criteria in the study aimed to assess its impact on liver histology. This consideration was particularly pertinent to the patient cohort, as many individuals seek medical attention only after a prolonged period of illness. It is essential to note that the natural progression of gallstones indicates that patients diagnosed with asymptomatic cholelithiasis have a 2% annual likelihood of developing symptoms [18]. Hence, there is a possibility of encountering patients who exhibit relative asymptomatic status despite harboring long-standing cholelithiasis.

Among various factors such as age, sex, smoking, substance abuse, hypertension, diabetes, presence of multiple stones, and BMI, patients with prolonged symptoms were found to be linked with abnormal liver histology. In this study, out of eight patients who had choledocholithiasis with obstructive symptoms such as jaundice, six patients (75%) underwent endoscopic retrograde cholangiopancreatography (ERCP) while two patients (25%) underwent common bile duct (CBD) exploration. Liver histology changes were present in six patients (75%), and all six patients had portal tract infiltration and cholestasis, and bile culture was positive in five patients. This observation aligns with the study findings of Patel G. et al. [3]. Consequently, the present study suggests a positive correlation between symptom duration and liver histological changes, indicating that longer durations of symptoms increase the likelihood of significant changes in liver biopsy. However, conflicting perspectives are noted in studies by other authors, such as Geraghty and Goldin [13], Morris-Stiff et al. [18], and Triger et al. [19], who contest the idea that symptom duration correlates with liver histology changes. Discrepancies in results could be attributed to variations in study types, inclusion criteria for study populations, and other factors. This study included the gallbladder for histopathological examination along with the liver edge for histopathological examination and bile aspirate for culture and sensitivity in one setting. Early intervention in these cases not only retards the unwanted effects but also prevents irreversible liver damage. One limitation of this study was that this study does not include other exogenous risk factors and genetic risk factors.

## Conclusions

Gallstone disease is associated with notable alterations in liver histology in about 36% of cases. There is a significant association between the duration of symptoms and changes in liver histology. Further studies are recommended with larger sample size to validate the findings of this study.
